# A Bayesian spline-augmented piecewise exponential model with spatial frailty for under-five mortality in Nigeria

**DOI:** 10.3389/fepid.2026.1826352

**Published:** 2026-07-16

**Authors:** Peter Enesi Omaku, Joseph Odunayo Braimah, Fabio Mathias Correa

**Affiliations:** 1 Department of Mathematical Statistics and Actuarial Sciences, University of the Free State, Bloemfontein, South Africa; 2 Department of Mathematics and Statistics, Ambrose Alli University, Ekpoma, Edo State, Nigeria

**Keywords:** INLA, latent Gaussian models, non-proportional hazards, penalized splines, spatial heterogeneity, time partition

## Abstract

**Introduction:**

The Cox proportional hazards (PH) model is widely used in time-to-event research, but its validity depends on the PH assumption, which can be violated in child mortality studies where hazards vary with age. Piecewise exponential models (PEMs) relax this assumption by partitioning follow-up time into intervals. However, standard formulations impose constant hazards within each interval and typically ignore spatial heterogeneity, limiting their usefulness for public health analyses in which geographical variation in risk is important. Existing extensions address non-proportional hazards or spatial dependence separately, but rarely combine a smooth baseline hazard, interval-level temporal heterogeneity, and structured spatial frailty within a single, computationally tractable Bayesian framework. This study proposes and applies such a framework to under-five mortality (U5M) in Nigeria.

**Methods:**

We formulated a Bayesian spline-augmented PEM incorporating a cubic-spline-smoothed baseline hazard, interval-specific Gaussian random effects, and a spatial frailty term with an intrinsic conditional autoregressive (ICAR) prior, within the latent Gaussian modeling framework of integrated nested Laplace approximation (INLA), with survival reformulated as a Poisson likelihood. The model was applied to U5M data for 103,439 children from the 2024 Nigeria Demographic and Health Survey (NDHS). Five nested model specifications (basic PEM, spline-augmented, spline plus interval effects, spline plus spatial effects, and the full spline-interval-spatial model) were compared across three sample sizes using DIC, WAIC, conditional predictive ordinates, posterior predictive checks, and calibration metrics.

**Results:**

The global test of the PH assumption was significant (χ^2^ = 898.66, *p* < 2 x 10^-16^), with all individual covariates significant at *p* < 0.05. Across all three sample sizes, the full spline-interval-spatial model achieved the lowest DIC and WAIC, the lowest CPO, and the highest posterior predictive correlation and calibration R^2^ among the five candidates, with the largest single reduction in DIC (approximately 17,884 units at n = 104,557) attributable to the addition of interval-specific random effects. In the fitted model, twin birth (HR = 2.82; 95% CrI: 2.62–3.03), breastfeeding status (HR = 0.45; 95% CrI: 0.42–0.49), and term delivery (HR = 0.47; 95% CrI: 0.41–0.53) had the largest effects on U5M, with additional protective effects for longer birth intervals and higher maternal education, and elevated risk in the North West (HR = 1.39) and North East (HR = 1.43) relative to the North Central region. Posterior spatial frailty estimates showed positive residual clustering concentrated in the North West and North East zones.

**Discussion:**

The results indicate that jointly modeling temporal and spatial heterogeneity yields the best-fitting and best-calibrated specification, although spatial frailty adds comparatively little once interval-level temporal effects are included, suggesting that residual heterogeneity in this setting is predominantly temporal rather than spatial. The Poisson-INLA formulation provides a computationally efficient alternative to MCMC-based spatial survival models, making it well suited to large-scale demographic surveys. From a public health perspective, the identified biological, maternal, and socioeconomic determinants point to the need for integrated interventions, while the persistence of unexplained spatial clustering after covariate adjustment indicates structural or contextual vulnerabilities in the northern zones in Nigeria.

## 1 Introduction

The Cox proportional hazards (PH) model (1) is a fundamental tool in survival analysis for time-to-event research. However, its accuracy hinges on the proportional hazards assumption, which is frequently violated in studies, especially those on child mortality, where hazard functions change with age (2). When this assumption is violated, standard Cox models can produce biased results. To address this, researchers often turn to time-partitioned models, such as the piecewise exponential model. These models assume constant hazards within specific time intervals but allow the hazards to vary between intervals (3). Spline-based methods, such as restricted cubic splines and penalized B-splines, provide a flexible way to model baseline hazards or log-cumulative hazard functions in parametric survival. However, a major limitation is that these models, which are based on partial or full likelihood estimation, generally do not account for spatial heterogeneity. This is a significant drawback for public health analyses, particularly in studies such as child mortality, where geographical variation is crucial (4, 5). Several statistical methodologies have been developed to address non-proportional hazards, including accelerated failure time (AFT) models (6), flexible parametric survival models utilizing restricted cubic splines (4, 7), and semiparametric Cox-based approaches (2). However, these frameworks have limitations when the goal is to jointly incorporate a smooth non-linear baseline hazard, interval-level temporal heterogeneity, and structured spatial dependence within a unified fully Bayesian framework (8, 9). Some packages supporting spatial survival modeling, including BayesX (10), mgcv (5), spBayesSurv (11), but the Markov Chain Monte Carlo (MCMC) inference, which becomes computationally prohibitive for large-scale datasets, and none natively combines penalized spline baseline hazards with ICAR spatial frailty in a single integrated model (12). In contrast, INLA (Integrated Nested Laplace Approximation) is an established framework for Bayesian latent Gaussian models, with support for spatial random effects through ICAR and SPDE models (8, 9).

To address those limitations, we propose an extension of the Bayesian survival modeling framework that uses a time-partitioning strategy to account for violations of the proportional hazards assumption. Cut-points are set at 6-month epochs throughout the 0–60 month follow-up window, consistent with Saroj (13), who justify this interval width as clinically meaningful for under-five mortality surveillance, and with standard practice in demography and child health research (3). Unlike traditional piecewise exponential models that impose constant hazards within each interval, we apply cubic spline smoothing to the baseline hazard function across the continuous time domain, capturing continuous and non-linear risk evolution independently of the interval partition. This spline-enhanced baseline is implemented within the latent Gaussian model framework of R-INLA, using a Poisson likelihood approximation.

To overcome the limitations of conventional survival models, this hybrid framework combines several key components. It begins by partitioning the follow-up period into intervals to handle violations of the proportional hazards assumption. Within these intervals, the model uses flexible cubic splines to estimate the baseline hazard and incorporates latent spatial effects using intrinsic conditional autoregressive (ICAR) models to account for geographical heterogeneity and provide a more comprehensive analysis. By expressing survival data in a Poisson observation framework (14, 15), the approach allows for fully Bayesian, interval, and spatially informed estimation of under-five mortality (U5M) risk while preserving temporal smoothness and accommodating local dependence structures.

Therefore, the present paper has the following objectives: The first objective is *methodological*: to propose, implement, and validate a Bayesian spline-augmented piecewise exponential model with spatial frailty within the R-INLA framework. The second objective is *applied*: to use the validated framework to analyze U5M risk factors and spatial patterns in Nigeria using data from the 2024 Nigeria Demographic and Health Survey (NDHS).

## 2 Methodology

### 2.1 Demographic and Health Survey (NDHS) dataset

The dataset used in this study was obtained from the 2024 Nigeria Demographic and Health Survey (NDHS), conducted by the National Population Commission (NPC) in collaboration with the National Malaria Elimination Program (NMEP) under Nigeria’s Federal Ministry of Health. From the total survey sample of 104,557 respondents, we assessed records from 103,439 individuals, focusing on factors influencing under-five mortality (U5M) nationwide. The subsample of 103,439 was derived by excluding children aged 5 years or older (60 months). The resulting dataset included children aged 1 to 60 months. The description of the variables is contained in Table 1.

**Table 1:** Description of variables for 2024 NDHS data for under-five mortality in Nigeria.

Covariates	Category and code
Status	Censored = 0; Dead = 1
Time	Age of child in months
Maternal Age at Birth (MAB)	Age of Mothers at birth in Years
Breastfeeding status (BF)	Never breastfed = 0; breastfed = 1
Preceding Birth Intervals (PBI)	<24 months = 0; 24–33 months = 1; More than 33 months = 2
Maternal Education Qualification (MEQ)	High Education = 3; Secondary = 2; Primary = 1; No education = 0
Wealth Index (WID)	Poorest = 0; Poorer = 1; Middle = 2; Richer = 3; Richest = 4
Region	NC = 0; NW = 1; NE = 2; SE = 3; SS = 4; SW = 5
Number of Antenatal Visits (NAV)	None = 0; 1–8 times = 1; 9 and above = 2
Duration of Pregnancy (DoP)	9 months = 0; More than 9 Months = 1
Sex of Child (sex)	Male = 0; Female = 1
Child Twin (Twin)	Singleton = 0; Twin = 1
Type of Place of Residence (TPR)	Urban = 0; Rural = 1
Use of Mosquito Net (UMN)	No = 0; Yes = 1
Contraceptive Use (CU)	No = 0; Yes = 1
Source of Drinking Water (SoDW)	Unimproved = 0; Improved = 1
Toilet Facility (TF)	Non and open air = 0; Pit = 1; Water system =2

*Source: Author’s Compilation.*

### 2.2 Model formulation

#### 2.2.1 Classical piecewise exponential model (PEM)

To accommodate violations of the proportional hazards (PH) assumption in time-to-event data (16), the follow-up time is partitioned into J disjoint intervals $\left[\tau_{\;j-1},\tau_{j}\right)$ for j=1,2,…,J, where $\tau_{0}=0$ and $\tau_{J}=\infty$. The hazard function is assumed to be constant within each interval but may vary across intervals. For the under-five mortality (U5M) analysis, we define the partition points τ={6,12,18,24,30,36,42,48,54} months, yielding the intervals: [0,6), [6,12), [12,18), [18,24), [24,30), [30,36), [36,42) [42,48), [48,54) and [54,∞). Saroj (13) study explicitly justifies the 6-month interval choice by indicating that the six-month time interval is highly critical for children till they reach five years of age.

The hazard function for an individual with covariate vector $x_{i}\in R^{p}$ at time t is given by:

$$h(t|x_{i})=\lambda_{j}\exp\;\left(x_{i}^{⊤}\beta \right),\;t\in [\tau_{\;j-1},\tau_{j}),\;j=1,\ldots ,J,\;i=1,\ldots ,n,$$ (1)

where $\lambda_{j}>0$ is the baseline hazard parameter for interval j, and $\beta \in R^{p}$ is the vector of regression coefficients capturing the effects of covariates on the hazard scale.

#### 2.2.2 Poisson likelihood reformulation for Bayesian computation

Following the standard likelihood equivalence established in survival analysis, the piecewise exponential model can be reformulated as a Poisson regression model. Let $d_{ij}\in \{0,1\}$ denote the event indicator for individual i in interval j, and let $t_{ij}>0$ denote the exposure time (time at risk) for individual i during interval j. Under the piecewise exponential assumption, the likelihood contribution for interval j is proportional to a Poisson likelihood with mean $\mu_{ij}=\lambda_{j}\exp\;(x_{i}^{⊤}\beta )t_{ij}$.

Formally, we specify:

$$d_{ij}|\mu_{ij}∼\text{Poisson}(\mu_{ij}),\;i=1,\ldots ,n,\;j=1,\ldots ,J,$$ (2)

with the log-linear predictor:

$$\log\;(\mu_{ij})=\alpha_{j}+x_{i}^{⊤}\beta +\log\;(t_{ij}),$$ (3)

where $\alpha_{j}=\log\;(\lambda_{j})\in R$ represents the log-baseline hazard for interval j, and $\log\;(t_{ij})$ is included as an offset term to account for differential exposure times.

#### 2.2.3 Spline-based baseline hazard smoothing

To induce smoothness in the baseline hazard while retaining the flexibility of the piecewise approach, we model the log-baseline hazard as a smooth function of the interval index j using cubic splines (4). We replace the interval-specific intercept $\alpha_{j}$ with a smooth function f(j):

$$\log\;(\mu_{ij})=f(j)+x_{i}^{⊤}\beta +\log\;(t_{ij}),$$ (4)

where f:R→R is a smooth function over the index of intervals. We specify f(j) using a cubic B-spline basis expansion:

$$f(j)=\sum_{k=1}^{K}\delta_{k}B_{k}(j),$$ (5)

where $\{B_{k}(\cdot ){\}}_{k=1}^{K}$ are B-spline basis functions of order 4 (cubic) defined over the domain [1,J], and $\delta =(\delta_{1},\ldots ,\delta_{K}{)}^{⊤}\in R^{K}$ is the vector of spline coefficients.

where $\{B_{k}(\cdot ){\}}_{k=1}^{K}$ are B-spline basis functions of order 4 (cubic) defined over the domain [1,J], and $\delta =(\delta_{1},\ldots ,\delta_{K}{)}^{⊤}\in R^{K}$ is the vector of spline coefficients. In this study, the spline basis was evaluated at the J=10 interval indices, with K=3 interior knots placed at the 25th, 50th, and 75th quantiles of the interval index distribution (5). The degree of smoothness was controlled by the precision hyperparameter $\tau_{\delta }$, estimated jointly with the other model parameters within R-INLA.

#### 2.2.4 Interval-level random effects (time-varying frailty)

To account for unobserved heterogeneity across time intervals not captured by the smooth baseline function, we include an interval-specific random intercept $u_{j}$:

$$\log\;(\mu_{ij})=f(j)+x_{i}^{⊤}\beta +\log\;(t_{ij})+u_{j}.$$ (6)

The random effects $\{u_{j}{\}}_{\;j=1}^{J}$ are assumed to be independently and identically distributed Gaussian:

$$u_{j}|\sigma_{u}^{2}\overset{i.i.d.}{∼}N(0,\sigma_{u}^{2}),\;j=1,\ldots ,J,$$ (7)

where $\sigma_{u}^{2}\geq 0$ is the variance parameter controlling the magnitude of interval-to-interval variability.

#### 2.2.5 Spatial random effects (Besag ICAR model)

To address geographical clustering in mortality risk, we incorporate a spatially structured random effect $\phi_{s}$ for the administrative unit s=1,…,S. Let s(i) denote the spatial unit corresponding to individual i. The model becomes:

$$\log\;(\mu_{ij})=f(j)+x_{i}^{⊤}\beta +\log\;(t_{ij})+u_{j}+\phi_{s(i)}.$$ (8)

For the spatial effects, we adopt the Besag intrinsic conditional autoregressive (ICAR) specification (8, 17). Under this model, the spatial vector $\phi =(\phi_{1},\ldots ,\phi_{S}{)}^{⊤}$ follows an ICAR prior:

$$\phi |\sigma_{\phi }^{2}∼\text{ICAR}(\sigma_{\phi }^{2}),$$ (9)

with joint prior density proportional to:

$$\pi (\phi |\sigma_{\phi }^{2})\propto {\left(\frac{1}{\sigma_{\phi }^{2}}\right)}^{(S-1)/2}\exp\;\left(-\frac{1}{2\sigma_{\phi }^{2}}\sum_{s∼t}(\phi_{s}-\phi_{t}{)}^{2}\right),$$ (10)

where s∼t denotes that regions s and t are adjacent, and $\sigma_{\phi }^{2}>0$ is the spatial variance parameter.

#### 2.2.6 Bayesian inference framework

##### 2.2.6.1 Likelihood Function

Let $D=\{d_{ij},t_{ij},x_{i},s(i)\}$ denote the observed data for i=1,…,n and j=1,…,J. Under the Poisson reformulation, the likelihood function for the parameter vector Θ=(δ,β,u,ϕ) is given by:

$$L(\Theta |D)=\prod_{i=1}^{n}\prod_{\;j=1}^{J}\frac{\mu_{ij}^{d_{ij}}e^{-\mu_{ij}}}{d_{ij}!},$$ (11)

where $\mu_{ij}=\exp\;(f(j)+x_{i}^{⊤}\beta +\log\;(t_{ij})+u_{j}+\phi_{s(i)})$.

##### 2.2.6.2 Prior Distributions

The prior distributions for the model parameters are specified as follows:

- **Spline coefficients:** For the spline coefficients δ, we assign a random walk prior of order 2 (RW2) to enforce smoothness (5, 18):
- **cubic spline** is a spline of order 4 (cubic polynomial pieces) with continuous first and second derivatives at the knots. It is the lowest-order spline where the human eye cannot typically detect the knots, making it the most popular choice in practical applications.

Let $a=t_{0}<t_{1}<t_{2}<\cdots <t_{K}=b$ be a sequence of knots dividing the interval [a,b] into K subintervals. A cubic spline f(x) satisfies:

1. On each subinterval $\left[t_{i-1},t_{i}\right]$, f(x) is a cubic polynomial:
  $$f(x)=a_{i}x^{3}+b_{i}x^{2}+c_{i}x+d_{i}.$$
2. At interior knots $t_{1},\ldots ,t_{K-1}$, the following continuity conditions hold:
  $$f(t_{i}^{-})=f(t_{i}^{+}),\;f^{\prime }(t_{i}^{-})=f^{\prime }(t_{i}^{+}),\;f^{\prime \prime }(t_{i}^{-})=f^{\prime \prime }(t_{i}^{+}).$$

These conditions ensure $f\in C^{2}[a,b]$, meaning the function, its first derivative, and its second derivative are all continuous everywhere on [a,b].

##### 2.2.6.3 Basis Representations for Cubic Splines

Any cubic spline can be written as a linear combination of basis functions. Two common representations exist:

The truncated power basis consists of:

- A global cubic polynomial: $1,x,x^{2},x^{3}$
- Truncated cubic basis functions at each knot $t_{k}$: $(x-t_{k}{)}_{+}^{3}$, where $\left(z{)}_{+}=\max(0,z\right)$.

Then any cubic spline can be written as:

$$f(x)=\beta_{0}+\beta_{1}x+\beta_{2}x^{2}+\beta_{3}x^{3}+\sum_{k=1}^{K}\beta_{3+k}(x-t_{k}{)}_{+}^{3}.$$

However, this representation is numerically unstable and difficult to penalize directly.

##### 2.2.6.4 B-Spline Basis

A more stable approach is the **B-spline basis** (basis splines), which has local support and better numerical properties. For cubic B-splines (order 4), the basis functions $B_{\;j,4}(x)$ satisfy:

- $\sum_{j}B_{\;j,4}(x)=1$ (partition of unity)
- Each $B_{\;j,4}(x)$ is non-negative and non-zero only on 4 consecutive intervals.

The spline is then expressed as:

$$f(x)=\sum_{\;j=1}^{n}\delta_{j}B_{\;j,4}(x)$$

where $\delta_{j}$ are the spline coefficients to be estimated.

##### 2.2.6.5 Penalizing the Cubic Spline to Enforce Smoothness

To prevent overfitting when many knots are used, we impose a smoothness penalty. The standard continuous penalty is the integrated squared second derivative:

$$\int [f^{\prime \prime }(x){]}^{2}dx.$$

For cubic splines, $f^{\prime \prime }(x)$ is piecewise linear. The penalty can be approximated by a quadratic form in the coefficients δ:

$$\int [f^{\prime \prime }(x){]}^{2}dx\approx \delta^{T}P\delta ,$$

where P is a penalty matrix.

##### 2.2.6.6 The Random Walk of Order 2 (RW2) Prior

For equally spaced knots with distance h=1 (scaling absorbed into the precision parameter), the second derivative of the spline at knot $x_{k}$ is approximated by the second-order finite difference:

$$f^{\prime \prime }(x_{k})\approx \delta_{k}-2\delta_{k-1}+\delta_{k-2}.$$

The **Random Walk of order 2 (RW2)** prior assumes these second differences are independently normally distributed with mean zero and precision $\tau_{\delta }$:

$$\Delta^{2}\delta_{k}=\delta_{k}-2\delta_{k-1}+\delta_{k-2}∼N(0,1/\tau_{\delta }),\;k=3,\ldots ,K.$$

This prior penalizes deviations from a straight line (since second differences are zero for a linear function). The joint prior density for $\delta =(\delta_{1},\ldots ,\delta_{K})$ is:

$$\pi (\delta |\tau_{\delta })\propto \tau_{\delta }^{(K-2)/2}\exp\left(-\frac{\tau_{\delta }}{2}\sum_{k=3}^{K}(\delta_{k}-2\delta_{k-1}+\delta_{k-2}{)}^{2}\right).$$

The exponent (K−2)/2 arises because there are K−2 independent second differences (the first two coefficients $\delta_{1},\delta_{2}$ are unrestricted in this improper prior). This prior is an **intrinsically Gaussian Markov random field (GMRF)** of order 2.

##### 2.2.6.7 Complete Cubic Spline Model with RW2 Prior

Combining the B-spline basis representation with the RW2 smoothness prior yields the complete Bayesian cubic spline model:

- **Mean function:**
  $$f(x)=\sum_{\;j=1}^{K}\delta_{j}B_{\;j,4}(x)$$
- **Prior on spline coefficients:**
  $$\pi (\delta |\tau_{\delta })\propto \tau_{\delta }^{(K-2)/2}\exp\left(-\frac{\tau_{\delta }}{2}\sum_{k=3}^{K}(\delta_{k}-2\delta_{k-1}+\delta_{k-2}{)}^{2}\right)$$
- **Hyperprior on precision (typical choice):**
  $$\tau_{\delta }∼\text{Gamma}(a,b)\;\text{or}\;\log\;(\tau_{\delta })∼N(0,\sigma^{2})$$

The complete representation of a cubic spline with an RW2 smoothness prior is therefore:

$$f(x)=\sum_{\;j=1}^{K}\delta_{j}B_{\;j,4}(x),\;\pi (\delta |\tau_{\delta })\propto \tau_{\delta }^{(K-2)/2}\exp\left(-\frac{\tau_{\delta }}{2}\sum_{k=3}^{K}(\delta_{k}-2\delta_{k-1}+\delta_{k-2}{)}^{2}\right)$$ (12)

where:

- $B_{\;j,4}(x)$ are cubic B-spline basis functions (order 4) defined on knots $t_{1},\ldots ,t_{K}$,
- $\delta =(\delta_{1},\ldots ,\delta_{K})$ are the spline coefficients,
- $\tau_{\delta }>0$ is the precision parameter (inverse variance) controlling smoothness: large $\tau_{\delta }$ forces nearly linear behavior,
- The prior is improper (rank deficiency 2) because linear functions have zero penalty.

##### 2.2.6.8 Implementation

In this study the Bayesian INLA software implement the RW2 prior as: $\delta |\tau_{\delta }∼N(0,\tau_{\delta }^{-1}R^{-})$,

where R is the precision matrix with nonzero entries only for |i−j|≤2, and $R^{-}$ denotes a generalized inverse. The cubic spline is then estimated by the posterior mean, yielding a smooth, flexible, and computationally efficient non-linear regression model.

##### 2.2.6.9 Regression coefficients:

For each regression coefficient $\beta_{p}$, p=1,…,P, we assign weakly informative Gaussian priors:

$$\beta_{p}\overset{i.i.d.}{∼}N(0,\sigma_{\beta }^{2}),\;\sigma_{\beta }^{2}=10^{3}.$$ (13)

#### 2.2.7 Interval frailty

The interval-specific random effects (19) follow:

$$u_{j}|\sigma_{u}^{2}\overset{i.i.d.}{∼}N(0,\sigma_{u}^{2}),\;j=1,\ldots ,J.$$ (14)

#### 2.2.8 Spatial frailty

The spatial random effects follow the Besag ICAR prior specified in Equation 10 (8, 17).

To account for spatial dependence among the S geographic regions, we assign an intrinsic conditional autoregressive (ICAR) prior (17) to the spatial random effects vector $\phi =(\phi_{1},\ldots ,\phi_{S}{)}^{⊤}$. The ICAR prior is an improper Gaussian Markov random field with density:

$$\pi (\phi |\sigma_{\phi }^{2})\propto {\left(\frac{1}{\sigma_{\phi }^{2}}\right)}^{(S-1)/2}\exp\;\left(-\frac{1}{2\sigma_{\phi }^{2}}\sum_{s∼t}(\phi_{s}-\phi_{t}{)}^{2}\right),$$

where s∼t denotes that regions s and t share a common border (are neighbors), the sum is taken over all unordered pairs of adjacent regions, and $\sigma_{\phi }^{2}>0$ is the spatial variance parameter controlling the degree of smoothing. The exponent (S−1)/2 reflects the rank deficiency of the precision matrix, which has nullspace spanned by the vector of ones; consequently, the prior does not constrain the overall mean of the spatial effects. To resolve this impropriety and ensure a proper posterior, we impose the sum-to-zero constraint $\sum_{s=1}^{S}\phi_{s}=0$ (9). The conditional formulation of this prior offers intuitive interpretation: for each region s with $d_{s}$ neighbors, the full conditional distribution is $\phi_{s}|\phi_{-s},\sigma_{\phi }^{2}∼N(d_{s}^{-1}\sum_{t\in \partial_{s}}\phi_{t},\;\sigma_{\phi }^{2}/d_{s})$, indicating that each spatial effect shrinks toward the mean of its neighbors with variance inversely proportional to the number of neighbors. Following standard practice in Bayesian spatial modeling (8, 20), we assign a weakly informative Inverse-Gamma hyperprior to the variance parameter: $\sigma_{\phi }^{2}∼\text{Inverse-Gamma}\;(a_{\phi },b_{\phi })$, with small shape parameters (e.g., $a_{\phi }=b_{\phi }=0.001$) to maintain approximate conjugacy while exerting minimal influence on the posterior.

The joint prior distribution of all unknown parameters is:

$$\pi (\delta ,\beta ,u,\phi ,\sigma_{u}^{2},\sigma_{\phi }^{2},\tau_{\delta })=\pi (\delta |\tau_{\delta })\;\pi (\tau_{\delta })\left(\prod_{\;p=1}^{P}\pi (\beta_{p})\right)\left(\prod_{\;j=1}^{J}\pi (u_{j}|\sigma_{u}^{2})\right)\pi (\sigma_{u}^{2})\;\pi (\phi |\sigma_{\phi }^{2})\;\pi (\sigma_{\phi }^{2})$$ (15)

##### 2.2.8.1 Posterior Distribution

By Bayes’ theorem, the joint posterior distribution is proportional to the product of the likelihood function and the joint prior distribution:

$$\begin{array}{rl} \pi (\delta ,\beta ,u,\phi ,\sigma_{u}^{2},\sigma_{\phi }^{2},\tau_{\delta }|D) & \propto L(\Theta |D)\times \pi (\delta ,\beta ,u,\phi ,\sigma_{u}^{2},\sigma_{\phi }^{2},\tau_{\delta })\\ & =\left[\prod_{i=1}^{n}\prod_{\;j=1}^{J}\frac{\mu_{ij}^{d_{ij}}e^{-\mu_{ij}}}{d_{ij}!}\right]\times \pi (\delta |\tau_{\delta })\pi (\tau_{\delta })\\ & \;\times \left[\prod_{\;p=1}^{P}N(\beta_{p}|0,\sigma_{\beta }^{2})\right]\times \left[\prod_{\;j=1}^{J}N(u_{j}|0,\sigma_{u}^{2})\right]\pi (\sigma_{u}^{2})\\ & \;\times \pi (\phi |\sigma_{\phi }^{2})\pi (\sigma_{\phi }^{2}). \end{array}$$ (16)

#### 2.2.9 Full model specification for U5M analysis

Our specification includes the **dual role of time intervals**. First, intervals define the partition for the piecewise exponential likelihood approximation, which includes the exposure offsets and event counts needed for the Poisson formulation. Second, we use interval-specific i.i.d. random effects $\epsilon_{i}∼N(0,\sigma_{\epsilon }^{2})$ to account for unobserved heterogeneity peculiar to each interval, such as seasonal epidemics or measurement mistakes. Combining interval random effects for overdispersion (14) and cubic splines for smooth baseline hazards (5) into a single framework with spatial frailty provides a useful modeling strategy that integrates well-established components. The cubic spline baseline functions on the continuous time scale and has its own knot placement that is independent of interval borders, allowing the smooth baseline to capture continuous death trajectories while the interval frailties absorb discrete, interval-level shocks. This decomposition gives various policy insights: the smooth baseline shows programming effectiveness, whereas interval frailties indicate periods that require special examination. This method also enables us to add spatial frailty using structured priors to account for the regional clustering of mortality risk. For the under-five mortality analysis in Nigeria, we specify the complete model with all covariates. Let $x_{i}$ denote the vector of covariates for individual i, comprising:

The full model is specified as: ?>

$$d_{ij}|\mu_{ij}∼\text{Poisson}(\mu_{ij}),$$ (17)

$$\begin{array}{rl} \log\;(\mu_{ij}) & =f(j)+\beta_{1}MAB_{ij}+\gamma_{1}SEX_{ij}+\gamma_{2}TWIN_{ij}+\gamma_{3}TPR_{ij}\\ & \;+\gamma_{4}TF_{ij}+\gamma_{5}SoDW_{ij}+\gamma_{6}PBI_{ij}+\gamma_{7}MEQ_{ij} \end{array}$$ (18)

$$\begin{array}{rl} & \;+\gamma_{9}BF_{ij}+\gamma_{10}CU_{ij}+\gamma_{11}UMN_{ij}+\gamma_{12}DoP_{ij}\\ & \;+\gamma_{13}NAV_{ij}+\gamma_{14}WID_{ij}+\gamma_{15}Region_{ij}+\log\;(t_{ij})+u_{j}+\phi_{s(i)}, \end{array}$$ (19)

where:

- $f(j)=\sum_{k=1}^{K}\delta_{k}B_{k}(j)$ is the spline-smoothed baseline hazard over time-split intervals
- $\beta_{1}$ is the regression coefficients for metrical covariate
- $\gamma_{1},\ldots ,\gamma_{15}$ are regression coefficients for categorical covariates
- $\log\;(t_{ij})$ is the offset term for exposure time
- $u_{j}|\sigma_{u}^{2}\overset{i.i.d.}{∼}N(0,\sigma_{u}^{2})$ are interval-specific frailty effects
- $\phi |\sigma_{\phi }^{2}∼\text{ICAR}(\sigma_{\phi }^{2})$ are spatial frailty effects for the 37 states in Nigeria

#### 2.2.10 Computational implementation via R-INLA

The proposed model is implemented within the Integrated Nested Laplace Approximation (INLA) framework (9). INLA provides deterministic approximations to posterior marginal distributions for latent Gaussian models, offering computational efficiency compared to MCMC methods.

The model components map to INLA as follows:

- **Spline component:** f(interval, model="rw2", constr=TRUE) for the RW2 prior on f(j), with the interval index as the covariate (J=10 distinct values) and K=3 interior knots, resulting in 7 basis functions. The sum-to-zero constraint constr=TRUE was imposed for identifiability
- **Interval frailty:** f(interval, model="iid") for $u_{j}$
- **Spatial frailty:** f(region, model="besag", graph=nigeria.graph) for the ICAR prior on ϕ; the besag model in R-INLA implements the intrinsic CAR specification of Besag et al. (17), which corresponds to the pure ICAR prior described in Equation 10, without an unstructured i.i.d. component.
- **Fixed effects:** Linear terms for β and γ coefficients
- **Offset:** E(time) for $\log\;(t_{ij})$

### 2.3 Data simulation and method’s validation

To validate the method, a simulation study was conducted, including repeated random subsampling from the original under-five mortality (U5M) survey dataset of 104,557 observations. The simulation was designed to assess the stability, predictive performance, robustness, and scalability of the proposed Bayesian piecewise-exponential modeling framework across varying sample sizes. Two large sample sizes, 10,000 and 50,000, were randomly selected without replacement from the entire survey dataset, along with the complete dataset (104,557). These samples were used to evaluate model behavior in moderate, large, and complete population scenarios, respectively. Repeated Monte Carlo replications were performed for each sample size to eliminate sampling variability and verify that the performance of competing models was assessed reliably.

The sampled dataset was initially preprocessed using Weibull-based stochastic imputation to resolve faulty or missing survival times, assuring the precisely positive survival durations required by the piecewise exponential modeling framework. The survival data were translated into counting-process form using the survSplit() algorithm, with follow-up time partitioned into 6-month intervals (6, 12,…, 54). This segmentation allowed the calculation of interval-specific baseline hazards while maintaining the time-to-event structure of the U5M data, and also served a second role by estimating the random effect. Each simulated dataset was fitted with five competing Bayesian piecewise exponential models: (i) the basic PEM; (ii) PEM with spline-smoothed baseline hazards (SPEM); (iii) SPEM with interval-level random effects; (iv) SPEM with spatial ICAR frailty effects; and (v) SPEM incorporating both interval random effects and spatial frailties. Bayesian inference was carried out using the Integrated Nested Laplace Approximation (INLA) method, which enables computationally efficient estimation of latent Gaussian survival models with complex hierarchical structures.

Model adequacy and prediction performance were evaluated using the Deviance Information Criterion (DIC), the Widely Applicable Information Criterion (WAIC), and the Conditional Predictive Ordinate (CPO). In addition, posterior predictive assessments were performed utilizing the posterior predictive root mean square error (PPC-RMSE) and the posterior predictive correlation between observed and fitted outcomes. Calibration performance was also assessed using calibration intercepts, slopes, and pseudo-R${}^{2}$ statistics generated from logistic calibration models.

To assess internal predictive validity, each collected dataset was divided into two subsets: training (80%) and testing (20%). Models were trained on the training data, and prediction accuracy was evaluated on the test data using root mean square error (RMSE). We assign a weakly informative Inverse-Gamma hyperprior to the variance parameter: $\sigma_{\phi }^{2}∼\text{Inverse-Gamma}(a_{\phi },b_{\phi })$, with small shape parameters ($a_{\phi }=b_{\phi }=0.001$) (21).

## 3 Results

This section is organized into two parts. Section 3.1 reports the results of the proposed model applied to the full NDHS dataset, including covariate effects, interval-specific hazards, and spatial frailty estimates. Section 3.2 presents the model comparison results from the simulation study.

### 3.1 Data analysis: proposed model applied to the 2024 NDHS

#### 3.1.1 Proportional hazards assumption

Table 2 reports the global test of the proportional hazards (PH) assumption. The global chi-square statistic was 898.66 ($p<2\times 10^{-16}$), and all individual covariates had statistically significant results (p<0.05). These results prove that the PH assumption is violated across the entire covariate set and confirm that a time-varying hazard structure is required for these data.

**Table 2:** Cox proportional hazards model diagnostic test results.

Covariate	chisq	df	*p*
Maternal age at birth	125.79	1	$<2\times 10^{-16}$
Sex	27.88	1	$1.3\times 10^{-7}$
Child Twin	162.27	1	$<2\times 10^{-16}$
Type of place of residence	63.29	1	$1.8\times 10^{-15}$
Source of drinking water	4.57	1	0.033
Breastfeeding status	73.13	1	$<2\times 10^{-16}$
Contraceptive use	23.22	1	$1.4\times 10^{-6}$
Use of mosquito net	31.01	1	$2.6\times 10^{-8}$
Toilet facility	196.98	2	$<2\times 10^{-16}$
Preceding birth intervals	8.04	2	0.018
Maternal education qualification	266.93	3	$<2\times 10^{-16}$
Duration of pregnancy	191.86	1	$<2\times 10^{-16}$
Number of antenatal visits	112.72	2	$<2\times 10^{-16}$
Wealth index	267.79	4	$<2\times 10^{-16}$
Region	185.72	5	$<2\times 10^{-16}$
**GLOBAL**	898.66	27	$<2\times 10^{-16}$

#### 3.1.2 Posterior fixed effects

**Figure 1:**
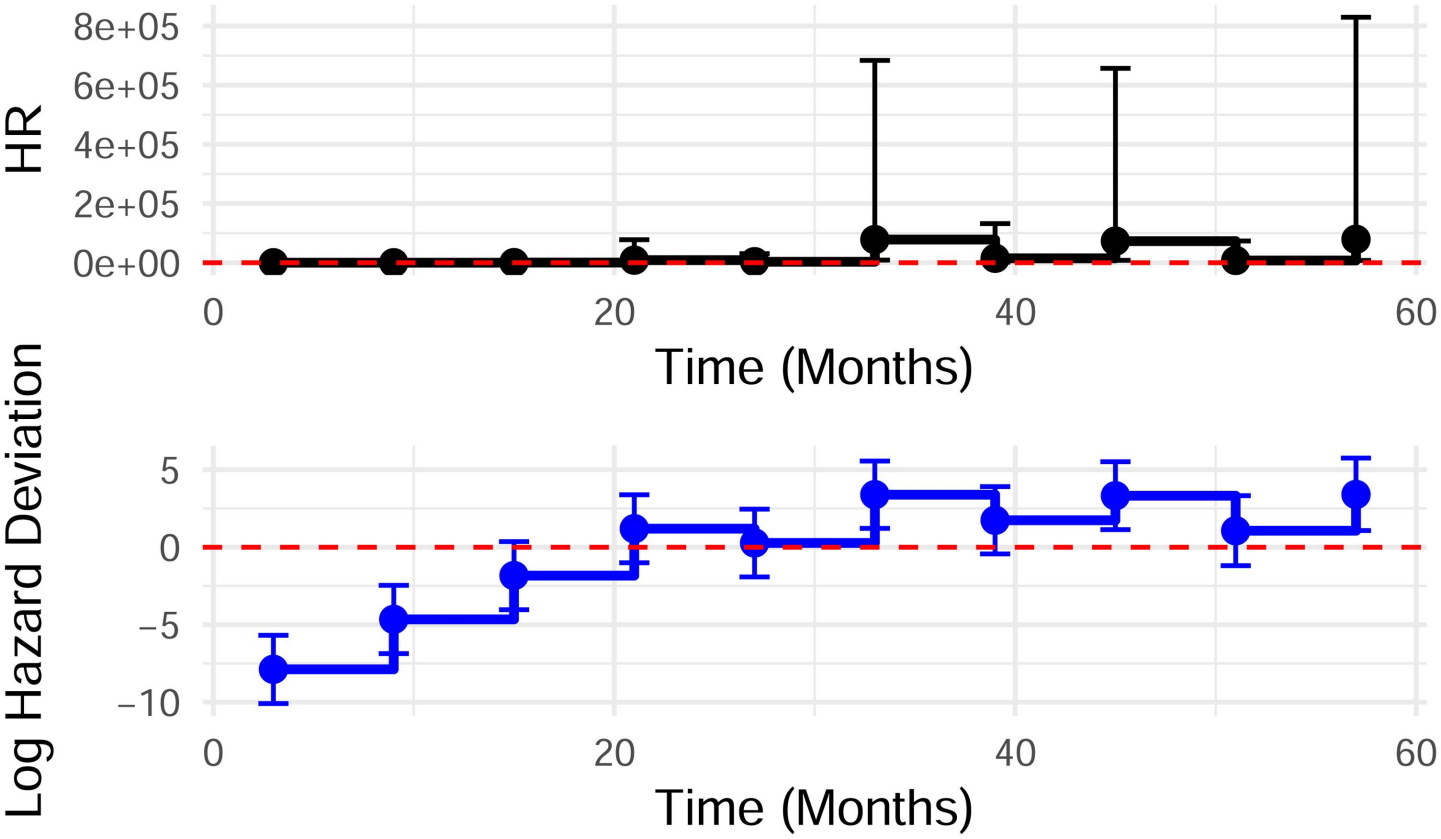
Interval effects in the piecewise exponential model. Top panel: constant baseline hazard estimator per interval. Bottom panel: random effect deviation per interval.

Table 3 presents the posterior fixed effects estimates, standard deviations, and hazard ratios (HR) with 95% credible intervals (CrI) from the spline-augmented piecewise exponential model (Model 3) fitted to the full NDHS dataset. The estimates identify twin birth (HR = 2.82), breastfeeding status (HR = 0.45), duration of pregnancy (HR = 0.47), and preceding birth interval (HR = 0.52 for >33 months) as the covariates with the largest effects on under-five mortality. Regional effects indicate elevated risk in the North West and North East relative to the North Central reference. The three spline baseline components (spline1-spline3) reflect the smooth log-hazard trajectory across age intervals (Figure 1).

**Table 3:** Posterior fixed effects estimates and hazard ratios from the spline-augmented piecewise exponential model.

Variable	Coefficient (β)	SD	HR	HR (2.5%)	HR (97.5%)
Intercept	6.121	1.126			
Maternal age at birth	0.002	0.001	1.002	0.999	1.005
Sex	−0.054	0.020	0.947	0.911	0.984
Twin	1.036	0.037	2.818	2.620	3.031
Type of Place of residence	0.229	0.027	1.257	1.191	1.327
Source of drinking water	−0.056	0.025	0.946	0.900	0.992
Breastfeeding Status	−0.797	0.037	0.451	0.419	0.485
Contraceptive use	−0.198	0.031	0.820	0.773	0.871
Use of mosquito net	0.109	0.025	1.115	1.063	1.171
Toilet facility 1	0.172	0.029	1.188	1.123	1.255
Toilet facility 2	0.150	0.041	1.162	1.070	1.260
Preceding birth interval 1	−0.196	0.022	0.822	0.787	0.858
Preceding birth interval 2	−0.646	0.031	0.524	0.493	0.558
Primary education	0.024	0.032	1.024	0.962	1.091
Secondary education	−0.166	0.034	0.847	0.792	0.907
Tertiary education	−0.571	0.061	0.565	0.501	0.637
Duration of Pregnancy 1	−0.757	0.065	0.469	0.412	0.533
Antenatal visits 1	−0.057	0.085	0.945	0.799	1.116
Antenatal visits 2	0.587	0.138	1.799	1.373	2.357
Wealth index 1	0.006	0.028	1.006	0.953	1.063
Wealth index 2	−0.190	0.035	0.827	0.773	0.885
Wealth index 3	−0.259	0.046	0.772	0.706	0.845
Wealth index 4	−0.277	0.063	0.758	0.670	0.858
North West	0.327	0.102	1.387	1.136	1.692
North East	0.360	0.090	1.433	1.202	1.709
South East	0.004	0.140	1.004	0.762	1.318
South West	−0.011	0.137	0.989	0.755	1.291
South South	−1.098	0.142	0.334	0.251	0.440
Spline baseline components (log-scale representation)					
spline1	−15.133	0.243	$2.67\times 10^{-7}$	$1.67\times 10^{-7}$	$4.30\times 10^{-7}$
spline2	−21.879	0.403	$3.15\times 10^{-10}$	$1.43\times 10^{-10}$	$6.93\times 10^{-10}$
spline3	−12.361	0.649	$4.28\times 10^{-6}$	$1.20\times 10^{-6}$	$1.53\times 10^{-5}$

#### 3.1.3 Interval-specific random effects and spatial frailty

Table 4 and Figure 1 show the interval-specific random effects. The mean log-random effects increase non-monotonically from −7.88 in the 0–6 month interval to positive values in later intervals, indicating periods of elevated residual hazard beyond the smooth baseline. The largest deviations occur around 30–36 and 42–60 months.

**Table 4:** Random effect estimates by time interval with hazard ratios.

interval_id	label	mean_log	mean_hr
1	0–6	−7.8786	1.00
2	6–12	−4.6575	25.06
3	12–18	−1.8316	422.85
4	18–24	1.1926	8,700.60
5	24–30	0.2730	3,469.02
6	30–36	3.3877	78,143.59
7	36–42	1.7365	14,989.04
8	42–48	3.3192	72,971.01
9	48–54	1.0591	7,613.58
10	54–60	3.3995	79,073.21

**Figure 2:**
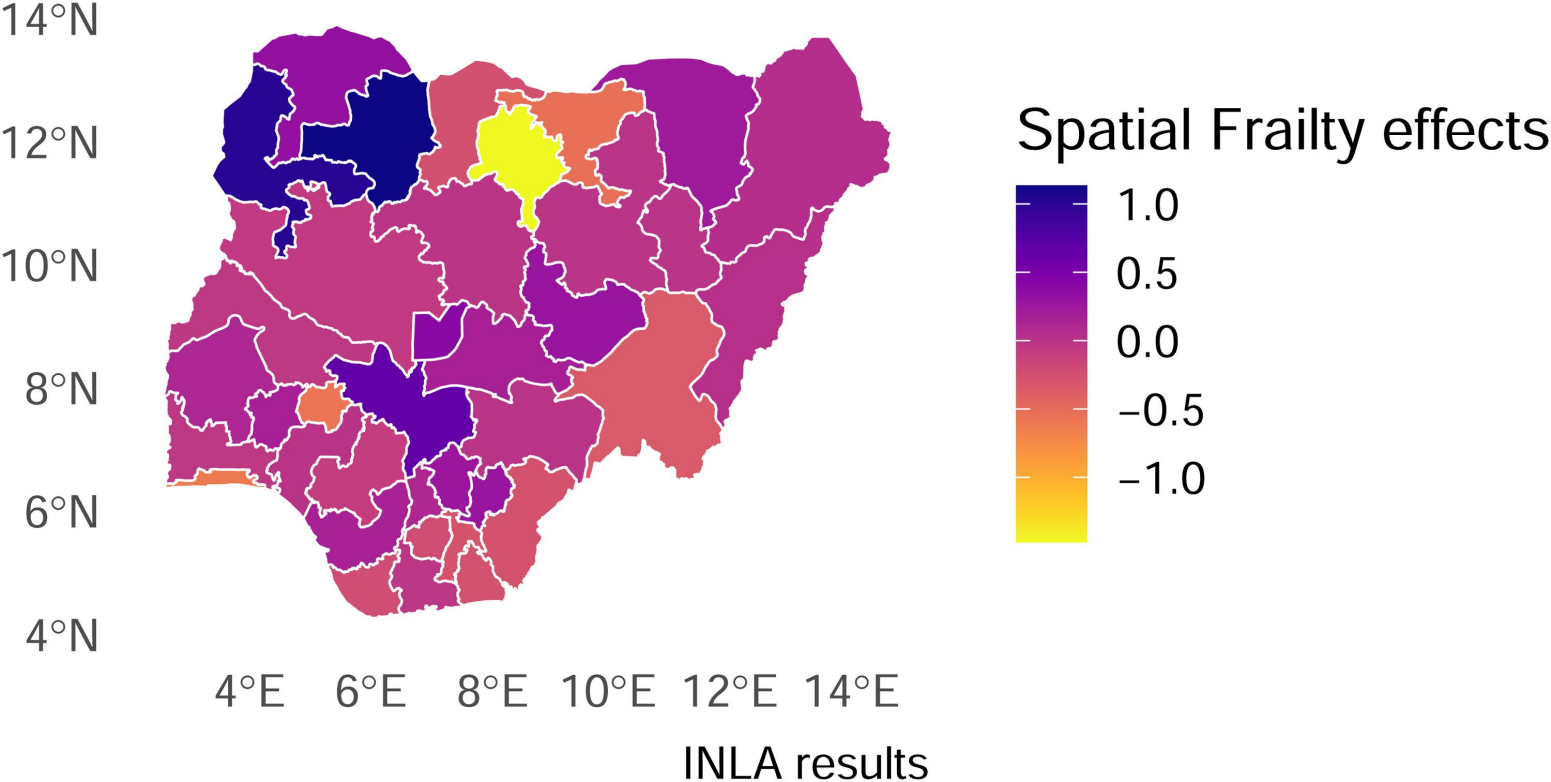
Posterior spatial frailty effects from the spline-augmented piecewise exponential model with interval and spatial random effects (Model 5).

Figure 2 shows the posterior spatial frailty estimates. Areas of higher positive frailty (darker shading) are concentrated in the far northeast and northwest, indicating higher-than-expected residual mortality beyond what is accounted for by the measured covariates. Southern and central states show predominantly negative frailties, indicating lower residual risk. The spatial pattern confirms the presence of unmeasured structural or socio-environmental disadvantages in the northern zones that are not captured by the included predictors.

### 3.2 Model comparison: simulation study

Table 5 summarizes the comparative performance of the five competing Bayesian piecewise exponential models across two sample sizes (n=10,000 and 50,000) and the full data (n=104,557). Across all sample sizes and all evaluation metrics, Model 5 (SPEM + Interval + Spatial) consistently achieves the lowest DIC and WAIC values, the lowest CPO, and the strongest posterior predictive correlation, while Test RMSE values remain stable across models. The addition of interval-specific random effects (Model 3 vs. Model 2) produces the single largest improvement in model fit, reducing DIC by approximately 1,680 units at n=10,000 and by approximately 17,884 units at n=104,557. The spatial-only extension (Model 4) improves upon Models 1 and 2 but falls short of the interval random effects models. The full Interval-Spatial model (Model 5) achieves the best $R^{2}$ across all sample sizes, indicating superior predictive reliability.

**Table 5:** Comparative performance of Bayesian piecewise exponential models across varying sample sizes.

Sample Size	Model	Mean DIC	Mean WAIC	Mean CPO	Mean PPC RMSE	Mean PPC COR	Calibration Slope	Calibration $R^{2}$	Test RMSE
10,000	Basic	9,123.972	9,124.207	0.154	0.186	0.131	7.470	0.039	0.189
10,000	+Splines	8,983.674	8,984.897	0.151	0.178	0.118	8.204	0.029	0.189
10,000	+Interval	7,303.624	7,301.483	0.123	0.175	0.170	10.612	0.048	0.189
10,000	+Spatial	8,958.409	8,959.045	0.151	0.178	0.124	9.000	0.032	0.189
10,000	+Interval_Spatial	7,274.909	7,270.839	0.122	0.175	0.176	11.562	0.051	0.189
50,000	Basic	46,292.083	46,292.040	0.157	0.189	0.126	7.041	0.036	0.186
50,000	+Splines	45,576.709	45,577.676	0.154	0.179	0.110	9.470	0.027	0.186
50,000	+Interval	37,383.600	37,380.892	0.127	0.179	0.157	12.445	0.042	0.186
50,000	+Spatial	45,317.847	45,318.720	0.153	0.179	0.118	9.873	0.030	0.186
50,000	+Interval_Spatial	37,108.825	37,104.159	0.126	0.179	0.163	15.070	0.045	0.186
104,557	Basic	96,926.888	96,926.790	0.157	0.190	0.128	6.831	0.037	0.184
104,557	+Splines	95,410.632	95,411.548	0.154	0.180	0.112	9.535	0.027	0.184
104,557	+Interval	78,176.674	78,173.806	0.127	0.179	0.159	13.757	0.043	0.184
104,557	+Spatial	94,789.379	94,790.496	0.153	0.179	0.121	10.078	0.031	0.184
104,557	+Interval_Spatial	77,527.554	77,522.856	0.125	0.180	0.166	20.474	0.045	0.184

## 4 Discussion

The proposed framework integrates three components within a single Bayesian R-INLA model: data-driven time partitioning (3, 22), spline-based hazard smoothing for continuous baseline risk estimation (4, 5, 18), and spatially structured ICAR frailty (8, 9, 20). The Poisson reformulation enables efficient Bayesian computation via R-INLA (12, 14, 23), while interval-level random effects (19, 24) capture unobserved temporal heterogeneity.

Alvares et al. (12) and Zhou et al. (11) used Bayesian survival models in INLA but rely on simpler parametric or frailty structures without spline baselines. Royston and Parmar (4) and Wood (5) develop spline-based survival models but within frequentist frameworks that do not accommodate spatial random effects. Adeyemi et al. (25) and Bamigbala and Ojetunde (26) examine spatial clustering of U5M in Nigeria using Bayesian spatial methods, but without the time-partitioned spline hazard structure proposed here. An advantage of the proposed framework is the semiparametric spatial frailty approaches. Methods such as those implemented in spBayesSurv offer considerable flexibility by allowing nonparametric baseline hazard models and accommodating spatial dependence via Bayesian hierarchical structures, typically estimated via MCMC. However, it often incurs substantial computational cost, which can become prohibitive for large datasets (11). Alternatively, the spline-augmented piecewise exponential model proposed here is based on a likelihood formulation that fits within latent Gaussian frameworks, enabling the use of the Integrated Nested Laplace Approximation (INLA) for fast and accurate inference. This leads to significant computational benefits while still allowing flexible hazard shapes to be specified through spline-based modeling and departures from proportional hazards. Therefore, the present framework constitutes a practical synthesis of these lines of work, combining their respective strengths within a computationally tractable hierarchical model.

### 4.1 Model comparison

The results in Table 5 show the evidence that incorporating both temporal and spatial random effects improves model fit and predictive performance. The violation of the proportional hazards assumption (Table 2), with a global chi-square of 898.66 ($p<2\times 10^{-16}$), confirms that a time-varying hazard structure is necessary and justifies the piecewise exponential approach with spline smoothing (5).

The basic PEM (Model 1) had the highest information criteria values and the lowest predictive correlation across all sample sizes. Incorporating spline-smoothed baseline hazards (Model 2) produces a moderate improvement in DIC and WAIC, consistent with findings in Royston and Parmar (4), but does not substantially alter predictive accuracy in isolation. The addition of interval-specific random effects (Model 3) produces the single largest reduction in DIC and WAIC, approximately 1,680 units at n=10,000 and 17,235 units at n=104,557, and improves the posterior predictive correlation, demonstrating that these random effects capture unobserved temporal heterogeneity that spline smoothing alone cannot absorb. The spatial-only extension (Model 4) improves upon Models 1 and 2 but falls short of Model 3, indicating that spatial dependence accounts for a smaller share of residual variance once the temporal structure is adequately represented (8). The full Interval-Spatial model (Model 5) achieves superior performance across all criteria and sample sizes, confirming that jointly modeling temporal and spatial heterogeneity is the preferred specification. The stability of results across sample sizes demonstrates the scalability and robustness of the framework.

### 4.2 Risk factors for under-five mortality in Nigeria

The application of the 2024 NDHS data reveals significant determinants of U5M in Nigeria, consistent with the literature on child health in sub-Saharan Africa (25, 27). The most informative findings are highlighted as follows.

#### 4.2.1 Biological risk factors

Twin birth (HR = 2.82; 95% CrI: 2.62–3.03) is the strongest risk factor, reflecting elevated mortality associated with lower birth weight and prematurity (2). Term delivery confers a 53% survival advantage over preterm (HR = 0.47; 95% CrI: 0.41–0.53).

#### 4.2.2 Maternal and reproductive factors

Breastfeeding status yields the largest protective effect (HR = 0.45; 95% CrI: 0.42–0.49), consistent with (2). Birth intervals of 24–33 months (HR = 0.82) and those over 33 months (HR = 0.52) confer stronger protection, supporting birth-spacing policies. Maternal education shows a dose-response gradient: secondary education reduces risk by 15%, tertiary by 44%, reinforcing the role of women’s education as a determinant of child survival (25).

#### 4.2.3 Socioeconomic and environmental factors

The wealth index shows a progressive protective gradient across higher quintiles. Improved drinking water sources confer a modest protective effect (HR = 0.95), consistent with the role of waterborne disease reduction. Place of residence (rural vs. urban: HR = 1.26) reflects underlying differences in healthcare access and environmental conditions.

#### 4.2.4 Unexpected associations

The positive association with mosquito net use (HR = 1.12) likely reflects confounding by malaria endemicity: families in highest-risk areas are more likely to use nets. The elevated risk for higher antenatal visit categories may similarly reflect confounding by high-risk pregnancies.

#### 4.2.5 Regional and spatial patterns

The North West (HR = 1.39) and North East (HR = 1.43) show elevated risk relative to the North Central reference, while South South shows a protective effect (HR = 0.33). The spatial frailty estimates in Figure 2 reveal additional residual clustering of unexplained mortality risk in the northeastern and northwestern zones after adjusting for all covariates, consistent with prior geospatial analyses (26, 27). The present analysis extends that literature by separating the component attributable to measured covariates from residual spatial heterogeneity, a distinction directly relevant for geographically targeted policy design.

#### 4.2.6 Temporal patterns

The interval-specific random effects (Table 4 and Figure 1) reveal non-monotonic positive deviations at 30–36 and 42–60 months beyond the smooth spline baseline, potentially reflecting seasonal epidemiological variation.

### 4.3 Limitations

This study has some limitations. First, the NDHS relies on retrospective birth history data, which may be subject to recall bias and misreporting, particularly for breastfeeding duration and healthcare utilization. Although these limitations are inherent to retrospective survey data, the NDHS remains the only nationally representative source with sufficient sample size and geographic coverage to support this type of analysis in Nigeria. Second, the time interval partition was selected on empirical grounds. Then, alternative structures could modify the estimated baseline hazard shape. Third, the cross-sectional design limits causal interpretation: the unexpected directions of some covariate effects (mosquito net use, higher antenatal visits) are plausibly explained by residual confounding that cannot be eliminated without longitudinal data or instrumental variable approaches. The simulation study was based on repeated random subsampling from the observed dataset rather than data generated from a known parametric model, which precludes evaluation of parameter recovery, estimation bias, or credible interval coverage in a controlled setting. Future work should complement these evaluations with fully synthetic simulation studies in which the ground truth is known.

## 5 Conclusion

This paper proposed and evaluated a Bayesian spline-augmented piecewise exponential survival model with spatial frailty implemented in R-INLA, and applied it to the analysis of under-five mortality in Nigeria using the 2024 NDHS data. The framework pursues two objectives: to assess the incremental contribution of spline smoothing, interval-specific random effects, and spatial frailty within a unified piecewise exponential specification, and apply it to identify risk factors and spatial patterns of U5M in Nigeria.

Among the five candidate models evaluated in this study, the full Interval-Spatial model (Model 5) achieved the best fit across all sample sizes and evaluation criteria, with the largest single improvement attributable to interval-specific random effects. These results indicate that, within the proposed framework, jointly temporal interval heterogeneity and structured spatial dependence yield better model fit than specifications that address only one of these components or neither. It should be noted, however, that the comparison was restricted to nested variants of the same piecewise exponential framework.

The analysis identified twin birth, short birth intervals, low maternal education, household poverty, and absence of breastfeeding as the principal risk factors, while breastfeeding, longer birth intervals, higher maternal education, and term delivery emerged as the strongest protective factors. The spatial frailty map shows residual clustering of mortality risk in the northeast and northwest, suggesting unmeasured structural vulnerabilities that require geographically targeted policy responses.

The proposed framework offers a computationally tractable and reproducible strategy for researchers working with large-scale complex survival data in public health settings, particularly where temporal non-proportionality and spatial heterogeneity are expected to coexist. Future work should include direct comparisons with established alternative survival modeling frameworks, such as accelerated failure time models, flexible parametric survival models, and semiparametric spatial approaches.

## Data Availability

The original contributions presented in the study are included in the article/Supplementary Material, further inquiries can be directed to the corresponding author/s.
